# Decreased breadth of the antibody response to the spike protein of SARS-CoV-2 after repeated vaccination

**DOI:** 10.3389/fimmu.2023.1157263

**Published:** 2023-04-03

**Authors:** Lydia Horndler, Pilar Delgado, Salvador Romero-Pinedo, Marina Quesada, Ivaylo Balabanov, Rocío Laguna-Goya, Patricia Almendro-Vázquez, Miguel A. Llamas, Manuel Fresno, Estela Paz-Artal, Hisse M. van Santen, Stela Álvarez-Fernández, Asunción Olmo, Balbino Alarcón

**Affiliations:** ^1^ Centro de Biología Molecular Severo Ochoa, Consejo Superior de Investigaciones Científicas (CSIC), Universidad Autónoma de Madrid, Madrid, Spain; ^2^ VITRO SA, Granada, Spain; ^3^ Instituto de Investigación Sanitaria Hospital 12 de Octubre (imas12), Madrid, Spain; ^4^ Empíreo Diagnóstico Molecular, Madrid, Spain; ^5^ Department of Immunology, Ophthalmology and ENT, Universidad Complutense de Madrid, Madrid, Spain

**Keywords:** SARS-CoV-2, S protein, flow cytometry method, vaccines, antibody breadth

## Abstract

**Introduction:**

The rapid development of vaccines to prevent COVID-19 has raised the need to compare the capacity of different vaccines in terms of developing a protective humoral response. Previous studies have shown inconsistent results in this area, highlighting the importance of further research to evaluate the efficacy of different vaccines.

**Methods:**

This study utilized a highly sensitive and reliable flow cytometry method to measure the titers of IgG1 isotype antibodies in the blood of healthy volunteers after receiving one or two doses of various vaccines administered in Spain. The method was also used to simultaneously measure the reactivity of antibodies to the S protein of the original Wuhan strain and variants B.1.1.7 (Alpha), B.1.617.2 (Delta), and B.1.617.1 (Kappa).

**Results:**

Significant differences were observed in the titer of anti-S antibodies produced after a first dose of the vaccines ChAdOx1 nCov-19/AstraZeneca, mRNA-1273/Moderna, BNT162b2/Pfizer-BioNTech, and Ad26.COV.S/Janssen. Furthermore, a relative reduction in the reactivity of the sera with the Alpha, Delta, and Kappa variants, compared to the Wuhan strain, was observed after the second boosting immunization.

**Discussion:**

The findings of this study provide a comparison of different vaccines in terms of anti-S antibody generation and cast doubts on the convenience of repeated immunization with the same S protein sequence. The multiplexed capacity of the flow cytometry method utilized in this study allowed for a comprehensive evaluation of the efficacy of various vaccines in generating a protective humoral response. Future research could focus on the implications of these findings for the development of effective COVID-19 vaccination strategies.

## Introduction

The rapid development of vaccines has enabled the prevention of severe symptoms caused by the SARS-CoV-2 virus responsible for COVID-19. This unprecedented speed in vaccine development has resulted in a significant decrease in COVID-19 deaths and hospitalizations in intensive care units (1). Vaccines are highly effective at preventing symptomatic disease, as demonstrated by clinical trials and clinical evidence. In Spain, the immunity reached by the widespread vaccination, and immunity due to previous infections, is likely responsible for the low number of deaths reported by the so-called fifth-wave by the end of July 2021; despite reaching similar number of infection cases to that of previous waves in the fall of 2020 and beginning of the year 2021 that resulted in a much higher number of deaths (https://covid19.who.int/region/euro/country/es). Different variants (SARS-CoV-2) have been identified since the first (COVID-19) infection appeared in December 2019. The vaccines that have been more commonly administered in Spain are the mRNA vaccines BNT162b2 (BNT) generated by BioNTech/Pfizer, mRNA-1273 by Moderna, and the adenovirus-based vaccines ChaAdOx1 nCov-19 (ChAd), and Ad26.COV2.S (Ad26) by Oxford University/AstraZeneca and Janssen/Johnsson&Johnsson, respectively. All vaccines demonstrated to be safe and protective in clinical trials (2–5), although reports of rare cases of thrombotic thrombocytopenia associated with both the ChAd and Ad26 vaccines have been published (5, 6). The advent of Spike (S) protein-based mRNA COVID-19 vaccines have significantly improved the COVID-19 pandemic, with both Pfizer-BioNTech (BNT162b2) and Moderna (mRNA-1,273) reporting 95% vaccine efficacy (1, 3).

Most countries have implemented vaccination schedules that consist of administering two initial doses of vaccines against SARS-CoV-2. In response to the emergence of new strains and the need for booster shots, several countries have recently added one to two additional doses of revaccination to their schedules (7). Vaccination induces neutralizing antibodies against key viral proteins, such as protein S, which effectively slows the spread of the virus and reduces the rate of severe morbidity and mortality (8). Recent research has shown that adenovirus-based COVID-19 vaccines have lower efficacy rates than mRNA vaccines. As a result, health authorities have recommended administering a third dose of mRNA vaccine as a booster to increase protection against the virus. This recommendation has been adopted in many countries, particularly for those who received the adenovirus-based vaccine as their primary vaccination. The third dose has been shown to increase protection against the virus and its variants, reducing the risk of severe illness and hospitalization (9, 10). The decreased sensitivity of neutralizing antibodies towards crucial viral proteins of SARS-CoV-2 variants such as Delta and Omicron has become a major issue, resulting in a possible reduction in vaccine efficacy. Although initial studies indicated positive outcomes in neutralizing these variants, recent research has emphasized the need for additional doses or booster shots to augment immunity against these variants. This decreased efficacy has also raised apprehension about the possibility of immune evasion by these variants, potentially circumventing vaccine-generated immunity (10). Consequently, ongoing research is concentrated on developing new vaccines and enhancing existing ones to offer better protection against these variants (11–13). The emergence of variants of concern (VOC) of SARS-CoV-2, particularly the highly transmissible Delta and Omicron variants, has posed a significant threat to vaccination strategies in Europe and the USA. The reduced efficacy of vaccines against these new variants has compromised their ability to prevent infection, transmission, and severe disease (10, 14, 15). Vaccines have been a critical tool in controlling the COVID-19 pandemic, but recent evidence suggests that fully vaccinated individuals can still become infected with SARS-CoV-2 and transmit the virus to others. This poses a significant challenge to achieve herd immunity and control the spread of the virus. In addition, the emergence of new VOC, such as the Delta and Omicron variants, highlights the potential for vaccinated individuals to harbor and transmit these variants (16). The emerging laboratory data indicate that vaccinated individuals show a significant reduction in neutralizing antibody response against the Omicron variant compared to the original strain of SARS-CoV-2 or the Delta variant (17). However, the neutralizing activity improves with the administration of booster doses. The emergence of the Delta and Omicron variants has led authorities in various countries to consider the administration of a third vaccine dose to the general population (7, 18). The capacity of the Delta variant to infect vaccinated individuals seems to correlate with its ability to escape neutralization by antibodies produced in response to vaccines (19). Thus, despite the significant loss of neutralizing antibody responses to Omicron, the preservation of spike-specific Fc receptor binding immunity may permit ongoing capture and clearance of the virus, providing persistent Fc-mediated protection against severe disease and death (20).

Although the third dose of vaccines can generate antibodies that partially neutralize Omicron and elicit cross-reactive T cell responses, early evidence suggests that the protection against viral infection offered by the booster dose is not fully effective and diminishes rapidly, highlighting the importance of continued monitoring and adaptation of vaccine strategies to address emerging variants (19, 21). Additionally, researchers are exploring other approaches, such as the development of new vaccines and the use of monoclonal antibodies, to combat emerging variants of the virus.

Serological tests are usually established by detecting the presence of viral antigen-specific IgG or IgM in the serum of individuals using recombinant fragments of the S or N proteins and tests based on ELISA or lateral flow assay (22–24). A disadvantage of those tests is that neutralizing antibodies are not directed against the N protein and that recombinant fragments of the S protein miss the quaternary structure of the protein trimer, which is the native form of the spike protein in the viral envelope. Therefore, part of the neutralizing antibodies directed against the native S trimer could be missed in serological tests based on the expression of recombinant proteins (23, 25). Recently, we have described a flow cytometry (FC) test that uses the cell line of hematopoietic origin Jurkat to express the native S protein of SARS-CoV-2 together with a truncated form of the human EGF receptor (huEGFRt) as a normalizing tool (26). The method allows the accurate determination of seropositivity and the measurement of antibody titers in sera from post-convalescent patients (26, 27). Here, we have used this FC method to compare the production of antibodies against the S protein in response to the prime immunization and the booster immunization of the four most common vaccines being used in Spain. In addition, we have used the multiplexed capacity of the method to simultaneously measure the reactivity of sera from vaccinated individuals with the S protein of the original Wuhan isolate versus the Alpha, Delta, and Kappa isolates as an example of VOC. Our results help to stir the debate about the convenience of the administration of more than a third dose of the same vaccines.

## Materials and methods

### Cells

The human T-cell line Jurkat clone E6-1 was acquired from ATCC (TIB-152) and was maintained in complete Roswell Park Memorial Institute medium (RPMI 1640), supplemented with 5% fetal bovine serum (FBS) (Sigma-Aldrich), 2mM L-*Gln*, 100U/ml penicillin and streptomycin in incubators at 5% CO_2_. Human embryonic kidney HEK293T cells (ATCC CRL-3216) were maintained in Dulbecco’s Modified Eagle Medium (DMEM) supplemented with 10% FBS, 2mM L-Gln, 100U/ml penicillin and streptomycin at 37°C/5% CO_2_. All cell lines were routinely tested for the presence of mycoplasma.

### Lentiviral vectors and Jurkat cell transduction

Spike (S) protein sequences were obtained from the Public Health England database (https://www.gov.uk/coronavirus) and synthesized by Eurofins Genomics (Ebersberg, Germany). For eukaryotic expression an epHIV-7-based lentiviral vector was used. It contains full-length S protein sequences of the Alpha (B.1.1.7), Delta (B.1.617.2) and Kappa (B.1.617.1) SARS-CoV-2 variants, followed by eGFP sequence, derived from the vector pEGFP-C1 (Addgene). Plasmid constructs were generated by the Gibson Assembly method (Gibson et al., 2009), using NEBuilder^®^ HiFi DNA Assembly Master Mix (New England Biolabs, Ipswich, MA, USA) according to manufacturer’s protocol.

The generation of Jurkat-S cells expressing the spike protein of the Wuhan variant has been previously described (26). Here, Jurkat cells were similarly transduced with lentivirus-containing supernatants, produced by transfected HEK-293T cells. Briefly, lentiviruses were obtained by the co-transfection of plasmids pCMV-dR (gag/pol) and pMD2.G (VSV envelope protein), using the jetPEI^®^ transfection reagent (Polyplus, Illkirch-Graffenstaden, France). Viral supernatants were collected 24 and 48 hours’ post-transfection. Polybrene (8 µg/mL) was added to the viral supernatants prior to transduction of Jurkat cells. A total of 5x10^5^ Jurkat cells were plated in 24-well flat-bottomed plates in a mix of 350µl RPMI and 350µl viral supernatant. Plates were centrifuged for 90 minutes at 2200 rpm and 32°C (1,5h/2200rpm/32°C) and left in culture for 24 hours. Transduced GFP+ or HuEGFRt+ cells were selected by FACS sorting 48 h later and expanded in culture. Wuhan and Alpha (B.1.1.7) variants were expressed in Jurkat CD3+ cells while Delta (B.1.617.2) and Kappa (B.1.617.1) variants were expressed in Jurkat CD3^–^ cells. The Jurkat CD3^–^ cells were generated in our lab using CRISPR/Cas9 system.

### Human sera

A total of 700 human sera were tested. A first set of 50 sera patients from Empireo laboratory obtained in the year 2020 after the initial wave of COVID-19 infections. Sera were assigned to different groups according to the clinical classification of disease severity, following a completed questionnaire by the donors/patients. The different groups were: Asymptomatic – from donors showing no symptoms; Mild – from patients experiencing 3 or more of the following symptoms: non-productive cough, hyperthermia, headache, odynophagia, dyspnea, asthenia, myalgia, ageusia, anosmia, cutaneous involvement; Moderate- indicating presence of 3 or more of the above symptoms plus gastrointestinal symptoms, or more than 3 of the above for 7 or more days; Moderate-Severe – meaning 3 or more of the above symptoms plus pneumonia; and Severe – developing pneumonia, requiring hospitalization and intubation. A second set of 52 serum samples was selected from the study “Immune response dynamics as predictor of COViD.19 disease evolution. Implications for therapeutic decision-making” from the Hospital Universitario La Princesa (HUP) and approved by the Research Ethics Committee (no. #4070). A third set of 250 sera were provided by nursery homes and VITRO employees. A fourth set of 40 serum samples were obtained from teachers and employees of a secondary school in Madrid (name not revealed to keep anonymity). And finally, a fifth cohort of 250 serum samples were obtained from capillary blood of volunteers working at the CBMSO in the period of January-July 2021, included in the study “*ACE2 as a biomarker with utility for identification of high risk population for SARS-CoV-2 infection and prognosis of evolution in COVID-19”* approved by the Research Ethics Committee (no. #2352). The ethical approvals for the laboratory use of samples applied to the current study. All participants provided written consent to participate in this study, which was performed according to the EU guidelines and following the ethical principles of the Declaration of Helsinki.

### Flow cytometry

For the study of relative binding of antibodies to the Alpha versus the Wuhan variants, a 1:1 mix of Jurkat-S(Wuhan) and Jurkat-S(Alpha) cells were incubated for 30 min on ice with 1:50 dilutions of human sera in phosphate-buffered saline (PBS), 1% bovine serum albumin (BSA) (Rockland Immunochemicals, Pottstown, PA, USA), 0.02% sodium azide. Cells were centrifuged for 5min at 900g and the pellet was resuspended in PBS-BSA buffer and spun again to eliminate excess immunoglobulins. Two additional washes were carried out. The cell pellet was finally resuspended in a 1:200 dilution of mouse anti-human IgG1 Fc-PE (SouthernBiotech, Birmingham, AL, USA) and a 1:200 dilution of Brilliant Violet 421™-conjugated anti-human EGFR Antibody (BioLegend, San Diego, CA, USA) in PBS-BSA, all antibodies were used under saturation conditions during staining. Samples were then washed, labeled with the viability dye 7-AAD and measured on a FACSCanto II flow cytometer (Becton-Dickinson), followed by data analysis using FlowJo v.10.3 software (BD Biosciences, Franklin Lakes, NJ, USA). For studying the four variants simultaneously, 1:1:1:1 mix of Jurkat-S(Wuhan), Jurkat-S(Alpha) Jurkat-S(Delta) and Jurkat-S(Kappa) cells were incubated as previously described, adding in a dilution 1:200 of the APC-labeled anti-human CD3 antibody (BD Ref.: 317317) to discriminate between CD3+ and CD3^–^ cells. The reactivity was calculated by first determining the individual ratio through the division of MFI IgG1 by MFI EGFR (26). Then, the ratio previously obtained for the new variants was divided by the ratio for the Wuhan strain.

### Confocal microscopy

Spike protein expression on the cell membrane was investigated by confocal microscopy. A total of 10^6^ cells expressing either of the VOCs were incubated with anti-SARS-CoV-2 spike antibody (GeneTex, Irvine, CA, USA; Cat No. GTX632604) at a 1:150 dilution in phosphate-buffered saline (PBS), 1% bovine serum albumin (BSA), and 0.02% sodium azide for 30 minutes at room temperature, followed by Alexa Fluor 555-labeled donkey anti-mouse IgG1 (ThermoFisher Scientific, Waltham, MA, USA, Cat No A-31570). Cells were then blocked with a 1:100 dilution of normal mouse serum for 15 minutes. After that, cells were labeled with anti-CD45-PE (BD, Cat No. 555483) at a 1:200 dilution for 30 minutes at room temperature. Samples were allowed to bind to poly-L-lysine-coated microscopy glass slides for 30 min and fixed for 10 min at room temperature with 4% paraformaldehyde. Cell nuclei were stained with 1 µg/ml DAPI in PBS for 5 min. Finally, samples were sealed with Prolong Antifade (Molecular probes). Observations were carried out on a LSM 710 laser scanning confocal microscope and image processing was performed using Zen software (both from Zeiss, Oberkochen, Germany).

### Neutralization assays

Lentiviral supernatants were produced from transfected HEK-293T cells as described previously (26). Briefly, lentiviruses were obtained by co-transfecting plasmids pCMV (gag/pol), pHRSINGFP and a truncated S envelope (pCR3.1-St) using the JetPEI transfection reagent (Polyplus Transfection). Viral supernatants were obtained after 48 hours post-transfection. Polybrene (8 µg/mL) was added to the viral supernatants prior to transduction of ACE2+HEK293T cells. A total of 35-50 x 10^3^ ACE2+HEK293T cells per p48 well were seeded the day before transduction. Serially diluted plasma was incubated with viral supernatant for 1 hour at 37 degrees prior to addition to the cells. Cells were left in culture for 48 hours, then were resuspended in PBS with 2% FBS and 5mM EDTA and fixed with 2% paraformaldehide. GFP+ cells were then analyzed on a FACSCalibur flow cytometer (Becton-Dickinson) and the data were analyzed with FlowJo software (BD).

### Statistics

All statistical analyses, as well as graph generation were carried out in GraphPad Prism 7.0 (GraphPad Software, San Diego, CA, USA). One-way analysis of variance (ANOVA) test was utilized for column analysis of the different groups. Statistical representation symbols mean: *p<0,05; **p<0,01; ***p<0,001; ****p<0,0001. Statistical parameters and the means +/- standard error of the mean (SEM) are represented in all graphs. Serum samples were received encoded from their sources and the researchers were blinded to their nature until all data analysis was finalized. Sample analysis was carried out in duplicate and all experiments were repeated a minimum of two times.

## Results

### Comparison of IgG1 anti-S titers in response to ChaAd, Moderna, BNT and Ad26 vaccines

**Figure 1:**
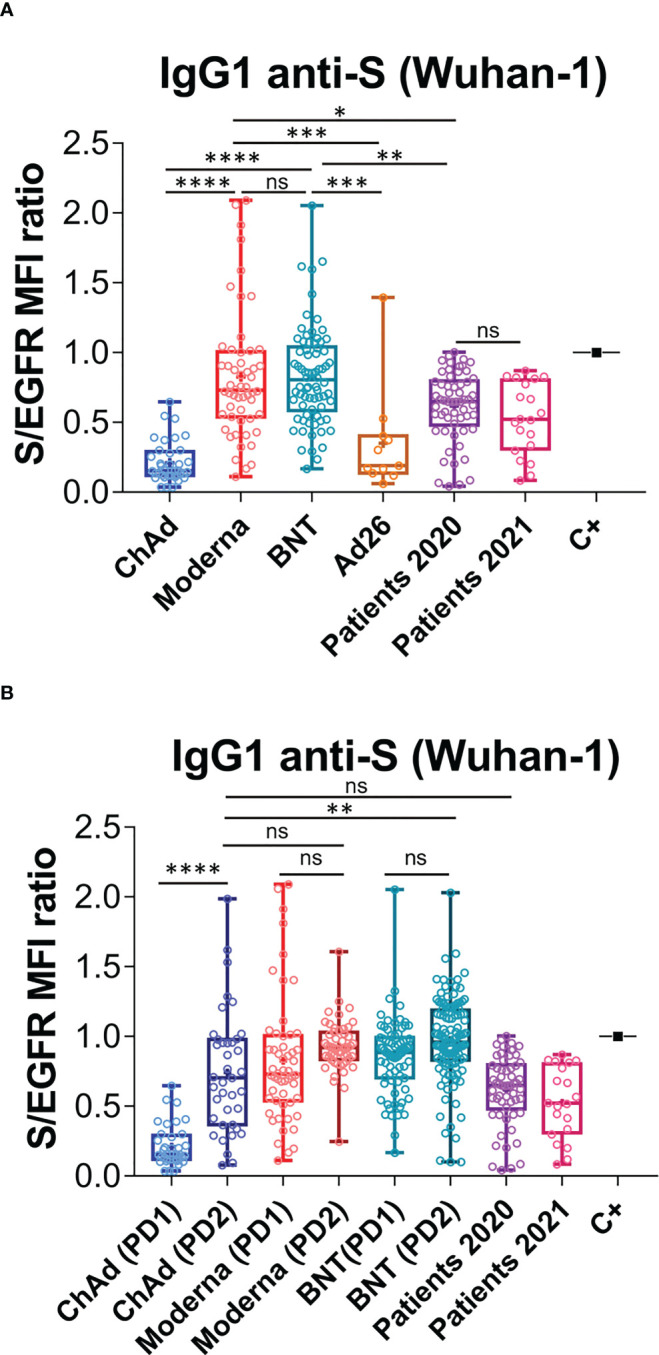
Generation of anti-S protein antibodies in response to the first and second priming-booster doses of different vaccines. **(A)** Generation of antibodies against the S protein of the Wuhan strain 3 weeks (BNT) or 4 weeks (Moderna, ChAd and Ad26) after the first priming dose of vaccine to previously uninfected individuals. The ratio of the mean fluorescence intensities (MFI) of anti-S staining and anti-EGFR staining was calculated for each serum sample and normalized to the value of the positive control (C+) sample. All sera were used at a 1:50 dilution. Box and whiskers plots to represent minimum and maximum values as well as the median, and the mean (the mean with a cross) is shown. *p<0.05; **p<0.01; ***p<0.001; ****p<0.0001; ns, not significant (one-way ANOVA test). **(B)** Generation of antibodies against the S protein of the Wuhan variant strain 3 weeks (BNT) or 4 weeks (Moderna, ChAd and Ad26) after the first, priming, dose of vaccine and 4 weeks (all vaccines) after the second, booster dose of vaccine. The ratio mean fluorescence intensities (MFI) of anti-S staining and anti-EGFR staining was calculated for each serum sample and normalized to the value of the positive control (C+) sample. All sera were used at a 1:50 dilution. Box and whiskers plots to represent minimum and maximum values as well as the median, and the mean (the mean with a cross) are shown. The number of samples for each vaccine is shown in the graph. **p<0.01; ****p<0.0001; ns, not significant (one-way ANOVA test).

We previously utilized a lentiviral vector to express the full spike “S” protein of SARS-CoV-2, containing the original Wuhan-1 sequence, followed by a truncated human EGFR protein (huEGFRt) and separated by a T2A self-cleaving peptide (26). This system allows the expression of two proteins from a monocistronic mRNA. We expressed this construct in the human leukemic cell line Jurkat by means of lentiviral transduction. We demonstrated that the S protein folded in its native form at the plasma membrane of Jurkat cells – electrophoretic separation in polyacrylamide gels under non-reducing conditions confirmed its trimeric structure. We also showed it was able to promote formation of syncytia between the Jurkat-S and ACE2^+^ HepG2 cells (26). Taking advantage that expression of the S protein is coupled to that of huEGFRt, we calculated a ratio of mean fluorescence intensities (MFI) of anti-S antibodies versus an anti-huEGFR antibody as direct estimation of the relative quantity of immunoglobulins against the S protein in sera, or other fluids, from different donors. A conversion between S/EGFR MFI ratios and international WHO standards in BAU/mL is shown in **Figure S1**. Using this system, we investigated the relative humoral response, in terms of IgG1 production, to the different vaccines administered in Spain from January to August 2021. Since the Ad26.COV2.S (Ad26) vaccine was initially planned to be given in a single dose unlike the other three ones (BNT162b2 [BNT], mRNA-1273 [Moderna], and ChaAdOx1 nCov-19 (ChAd)], we first compared the titer of antibodies elicited in vaccinated healthy individuals 3-4 weeks after the first dose. We found that ChAd (n=37) induced lower titers of anti-S IgG1 antibodies than Moderna (n=54) and BNT (n=75) (**Figure 1A**). However, the titers elicited in response to ChAd and Ad26 (n=11) were not significantly different. Compared to a cohort of patients recovered from SARS-CoV-2 infection between March and May 2020 (https://covid19.who.int/region/euro/country/es) (Patients 2020, n=58, **Figure 1A**), vaccination with one dose of ChAd led to significantly lower antibody production (*P*<0.0001). By contrast, a single dose of either BNT or Moderna induced higher titers than those of Patients 2020. Unfortunately, the number of samples in the Ad26 group was insufficient to establish a significant comparison with the Patients 2020 cohort. Altogether, these data suggest that the two RNA vaccines (BNT and Moderna) are better at inducing IgG1 anti-S antibodies than the two adenovirus-based vaccines (Ad26 and ChAd).

We next compared the titers of anti-S IgG1, generated in response to a priming and a booster dose of the ChAd, Moderna and BNT vaccines. The second dose of ChAd vaccine (PD2; n=39) substantially increased antibody production to a level comparable to that of Patients 2020 and to that of individuals vaccinated with two doses of Moderna (PD2; n=52; **Figure 1B**). Nonetheless, antibody titers elicited by two doses of BNT (PD2, n=119) were still significantly higher than those generated in response to two doses of ChAd (**Figure 1B**). Interestingly, the titers of IgG1 achieved upon administration of a booster dose (PD2) of either Moderna or BNT, were not significantly higher than those produced after the priming dose (PD1, **Figure 1B**). These results do not argue against booster doses of the Moderna and BNT vaccines, as their effects on the humoral immunity, like improving the memory response or the duration of protection have not been evaluated.

### IgG1 titers against the S protein of the Alpha (B.1.1.7) VOC produced after priming-booster vaccination

**Figure 2:**
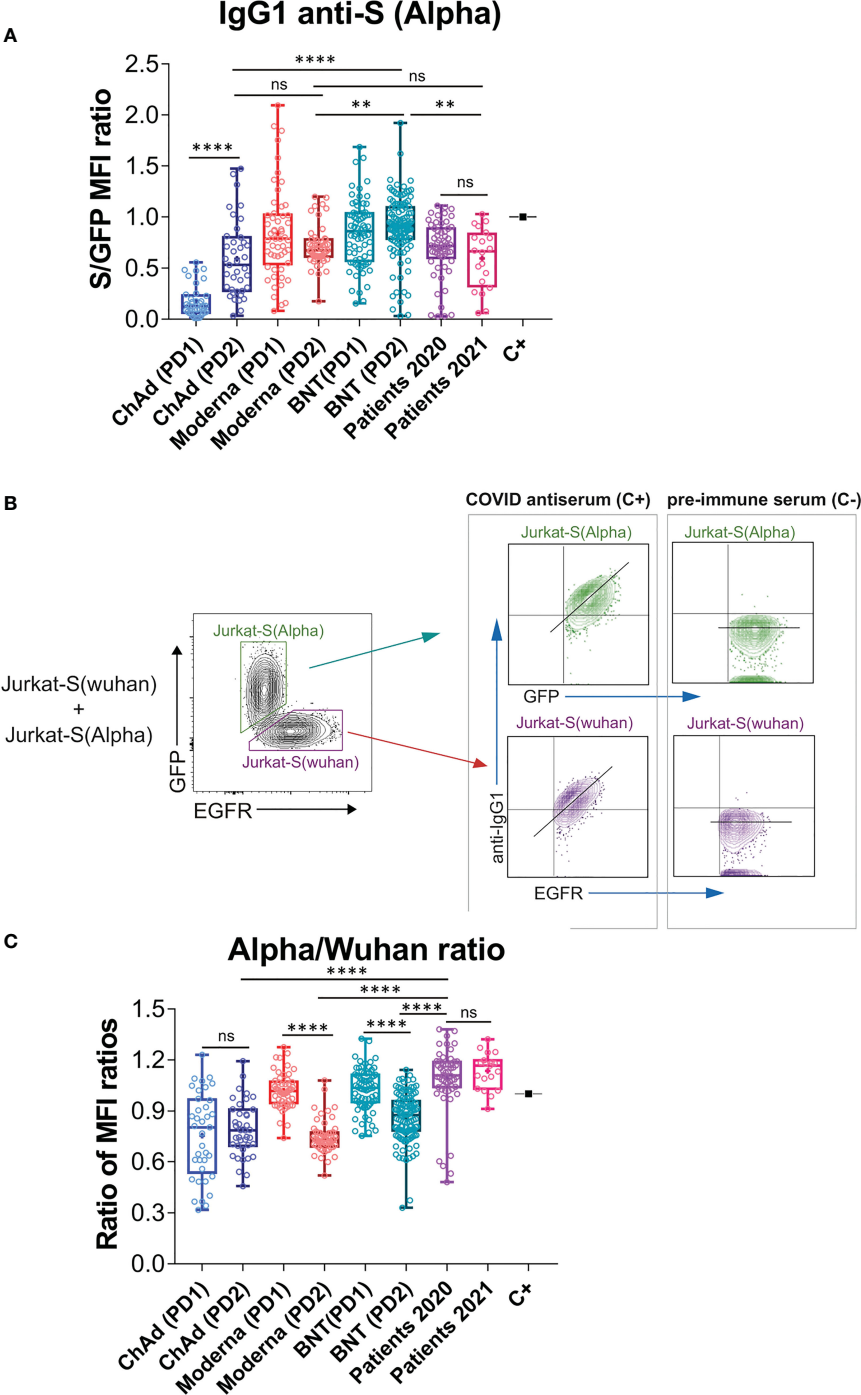
Effect of vaccination on the relative recognition of the Alpha and Wuhan variants by sera of vaccinated individuals. **(A)** Antibody generation against the S protein of the Alpha variant was evaluated 3 weeks after the first dose of BNT vaccine and 4 weeks after the first dose of Moderna, ChAd, and Ad26 vaccines. The evaluation was also done 4 weeks after the second booster dose for all vaccines. The ratio of mean fluorescence intensities (MFI) of anti-S staining and GFP staining was determined for each serum sample and normalized to the positive control (C+) sample. All sera were used at a 1:50 dilution. Box and whisker plots were used to represent the minimum and maximum values, as well as the median and mean (the mean with a cross). The graph shows the number of samples for each vaccine. The statistical significance of the results was determined using one-way ANOVA test, with ** indicating p<0.01, **** indicating p<0.0001, and ns indicating not significant. **(B)** Gating strategy to measure binding to the Wuhan and Alpha variants. Jurkat-S(Wuhan) and Jurkat-S(Alpha) cells were mixed in 1:1 ratio and incubated with either a 1:50 dilution of a positive control serum (C+) from a patient of the first wave in 2020 or a negative control serum (C-) obtained before the COVID-19 pandemics. Cells were subsequently incubated with mouse anti-human IgG1-PE and mouse anti-human EGFR-BV421 antibodies. After incubation, cells were gated according to lymphocyte FSC-A and SSC-A and subsequently on 7-AAD negative cells (7-AAD+ are dead cells). Live cells were subsequently gated on the FL1 channel (for GFP+ cells) and on the FL7 channel (huEGFRt+ cells). Both GFP+ and huEGFRt+ cells were separately analyzed for the fluorescence intensity of anti-IgG1 (S protein, FL2) versus GFP (for Jurkat-S(Alpha)) or for the fluorescence intensity of anti-IgG1 (S protein, FL2) versus EGFR (for Jurkat-S(Wuhan)). A diagonal lane is plotted on the bicolor contour plots corresponding to the C+ staining to indicate a relationship between S protein expression and either GFP or huEGFRt. **(C)** Relative reactivity of the sera tested in **(A)** against the Alpha and Wuhan variants measured according to the (S/GFP ratio)/(S/EGFR ratio) and plotted individually for each serum donor. ****p<0.0001; ns, not significant (one-way ANOVA test).

The flow cytometry Jurkat-S method can be easily multiplexed to measure simultaneously generation of different antibodies to a single protein or to several proteins (26). We decided to use this property to compare antibodies that bind the S protein of the Wuhan strain to the S protein of the Alpha (B.1.1.7) VOC. We suggest that this comparison might be relevant given that the present vaccines were all generated using the Wuhan strain. The S protein sequence of the Alpha VOC differs in just 7 positions from that of the Wuhan strain (**Figure S2**). The Alpha VOC became dominant in Spain during the spring of 2021 (https://theconversation.com/the-uk-variant-is-likely-deadlier-more-infectious-and-becoming-dominant-but-the-vaccines-still-work-well-against-it-156951) and consequently we included in this and previous experiments a cohort of sera from patients recovered from SARS-CoV-2 infection between March and May 2021 (Patients 2021, n=20, **Figures 1**, **2**).

In order to detect the presence of antibodies against the S protein of the Alpha VOC, we cloned the Spike sequence of the variant (**Figure S2**) into the same lentiviral vector used to generate the original Jurkat-S cells (26), but replaced the huEGFRt reporter by EGFP, and generated Jurkat cells stably expressing the S protein of the Alpha variant, followed by EGFP. Using this new Jurkat-S cells (henceforth designated as Jurkat-S[Alpha]) we could measure the titers of IgG1 antibodies specific for the S protein of the Alpha VOC. A positive control serum from a patient recovered from infection during the first wave of COVID-19 in March 2020 was titrated in both Jurkat-S[Wuhan] and Jurkat-S[Alpha] generating very similar results (**Figure S3**). We used a non-saturating 1:50 dilution of all sera, as in **Figure 1**, to compare the response to prime-boost vaccination in terms of generation of IgG1 antibodies capable of binding the Wuhan and Alpha variants simultaneously. We found similar results to those for the Wuhan strain alone (compare **Figure 2A** with **Figure 1B**), namely that the second dose of ChAd significantly increased the titer of antibodies compared to the first dose and that the second dose of Moderna and BNT did not lead to a significant increase of IgG1 anti-S titers compared to the first, priming dose (P=0.43 and P=0.92, respectively; One-way ANOVA test).

Taking advantage of the distinct markers used for Jurkat-S(Wuhan) and Jurkat-S(Alpha) cells (EGFR and EGFP, respectively), we could mix equal numbers of both cell lines and incubate the cell mixture with dilutions of sera from patients recovered from infection with SARS-CoV-2 or vaccinated. The analysis by flow cytometry allowed to gate on the eGFP^+^ cells to calculate the ratio of [fluorescence anti-S/GFP fluorescence] as a measurement of the titer of antibodies of the IgG1 isotype against the S protein of the Alpha variant (**Figure 2B**). Simultaneously, we could gate on the EGFR+ cells to calculate the ratio of [fluorescence anti-S/fluorescence anti-EGFR] as a measurement of the titer of antibodies of the IgG1 isotype against the S protein of the Wuhan strain. The combination of Jurkat-S(Wuhan) and Jurkat-S(Alpha) cells in the same experiment allowed us to calculate a ratio of ratios (S/GFP vs S/EGFR) that serves to compare sera from vaccinated volunteers in terms of their relative reactivity with the S protein of the Wuhan and Alpha VOC. The mixing of both cell types in this setting would allow the S protein of Alpha to compete with the S protein of Wuhan in order to trap the highest affinity IgG1 antibodies. Therefore, this assay would theoretically serve to detect relative binding to one or another variant by antibodies that have undergone affinity maturation, a signature of secondary humoral response. Interestingly, the second dose of Moderna and the second dose of BNT caused a relative decrease in the reactivity of antibodies with the Alpha VOC in comparison with the Wuhan-1 isolate (**Figure 2C**). The second dose of ChAd did neither increase, nor decrease the relative recognition of the S protein of Alpha compared to that of the Wuhan variant.

Comparison of the relative reactivity of sera from vaccinated individuals with those of people recovered from infection in 2020 and 2021, showed that antibodies produced in response to the second dose of either of the three vaccines were relatively less reactive to the Alpha strain than those produced by natural infection not only in 2021 but also in 2020 (**Figure 2C**). Those results suggest that the booster immunization with a second dose of the three vaccines, based on the sequence of the Wuhan strain, might compromise the reactivity of antibodies towards emerging VOCs of SARS-CoV-2.

### General decrease of relative reactivity against VOCs after booster vaccination

**Figure 3:**
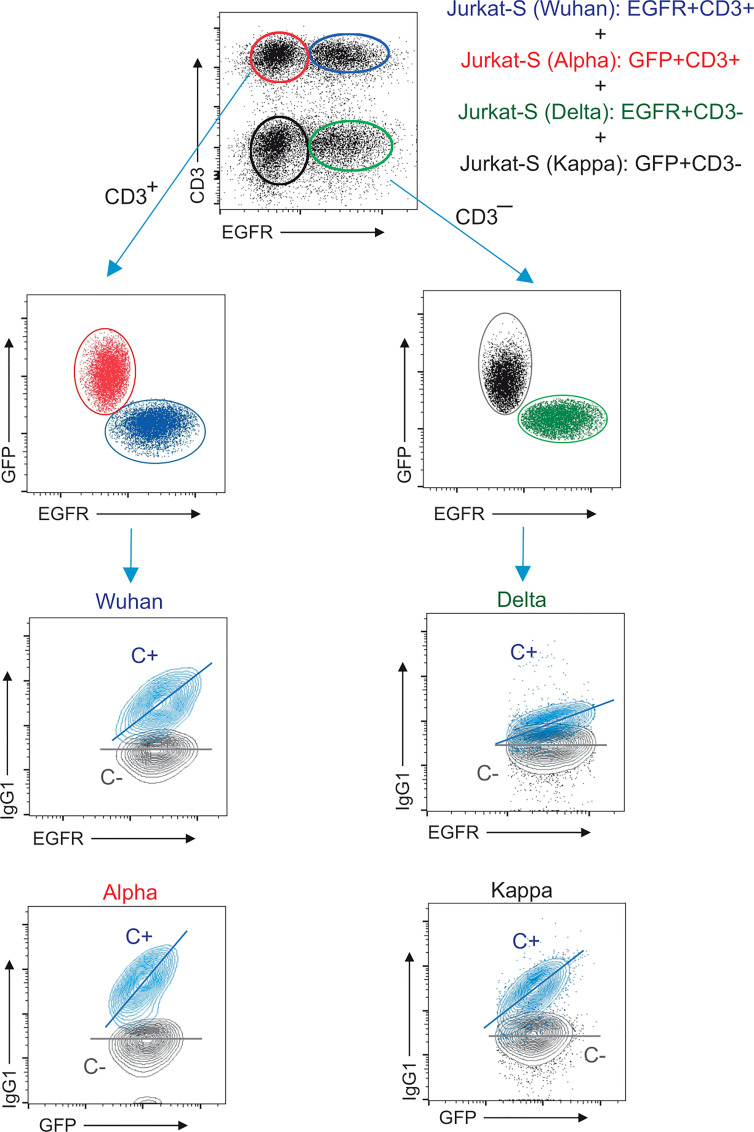
Multiplexed method to measure the reactivity of IgG1 antibodies with the S protein of the Wuhan, Alpha, Delta and Kappa variants. Gating strategy to measure binding to the Wuhan, Alpha, Delta and Kappa variants. Jurkat-S(Wuhan), Jurkat-S(Alpha), Jurkat-S(Delta) and Jurkat-S(Kappa) cells were mixed in 1:1:1:1 ratio and incubated with either a 1:50 dilution of a positive control serum (C+) from a patient of the first wave in 2020 or a negative control serum (C-) obtained before the COVID-19 pandemics. Cells were subsequently incubated with mouse anti-human IgG1-PE, mouse anti-CD3-APC and mouse anti-human EGFR-BV421 antibodies. After incubation, cells were gated according to lymphocyte FSC-A and SSC-A and subsequently on 7-AAD negative cells (7-AAD+ are dead cells). Live cells were subsequently gated on the FL1 channel (for CD3+ cells) and on the FL7 channel (huEGFRt+ cells). This allowed to distinguish four populations of cells: EGFR+CD3+ (Jurkat-S[Wuhan], EGFR^–^CD3+ (Jurkat-S[Alpha]), EGFR+CD3^–^ (Jurkat-S[Delta]) and EGFR^–^CD3^–^ (Jurkat-S[Kappa]). The cells were split between CD3+ and CD3^–^ cells and subsequently analyzed according to EGFR and GFP expression (FL7 and FL1 channels). The four cell populations were separately analyzed for the fluorescence intensity of anti-IgG1 (S protein, FL2) versus GFP (for Jurkat-S[Alpha] and Jurkat-S[Kappa]) and for the fluorescence intensity of anti-IgG1 (S protein, FL2) versus EGFR (for Jurkat-S[Wuhan] and Jurkat-S[Delta]). A diagonal lane is plotted on the bicolor contour plots corresponding to the C+ staining to indicate a relationship between S protein expression and either GFP or huEGFRt.

To determine if the decrease in anti-S (Alpha) relative reactivity after a second immunization was also present in more recent VOCs of SARS-CoV-2, we modified the Jurkat-S system to measure IgG1 binding to two more virus variants – Delta (B.1.617.2) and Kappa (B.1.617.1), which had become the dominant variant in Europe by the summer of 2021. In order to be able to distinguish Jurkat cells expressing the S protein of the Delta and Kappa variants from the Wuhan and Alpha ones using the same lentiviral vectors with the EGFR and EGFP markers, we transduced a Jurkat CD3-deficient cell line, that was previously generated in our lab by CRISPR/Cas9 deletion of the CD3ζ subunit of the TCR. Those CD3^–^ Jurkat cells were transduced with the lentiviral vectors co-expressing the S protein from Delta VOC coupled to EGFR or from Kappa VOC with EGFP. Using this strategy, we could mix the four Jurkat-S cell lines and incubate them simultaneously with serum samples to later measure IgG1 binding to the 4 VOCs together using CD3, EGFR and GFP markers (**Figure 3**). **Figure 3** illustrates how the positive control serum and a negative control serum stain the four VOCs. A positive serum containing IgG1 binding to any of the 4 VOCs produces a diagonal, whereas a negative serum produces a flat line (**Figure 3**). The S/EGFR and S/GFP MFI ratios produced by the positive control serum for those two Jurkat-S(Delta) and Jurkat-S(Kappa) variants were reduced in terms of absolute numbers, compared to the values generated with the MFI ratios for the Wuhan and Alpha VOCs. The differences in MFI ratio numbers could be intrinsic to the use of CD3^–^ versus wild type Jurkat cells. However, the titration of the positive control serum produced very similar results for the 4 variants (**Figure S3**), suggesting that the method is robustly detecting antibodies against the 4 VOCs. As a control for correct expression of the 4 variants we analyzed the distribution of recombinant S protein *via* confocal microscopy. Results showed that in all of the 4 variants tested the spike S protein was actively expressed at the plasma membrane (**Figure S4**).

**Figure 4:**
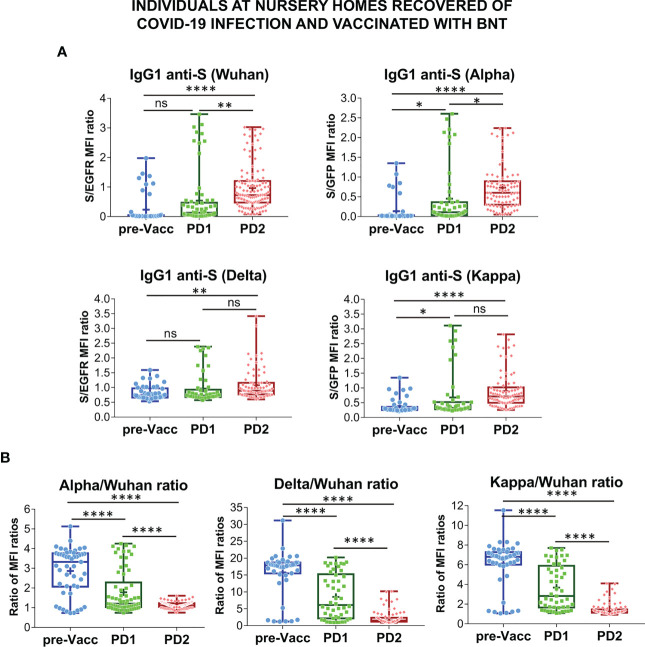
Follow-up of the antibody response to vaccination in nursing homes. **(A)** Generation of antibodies against the S protein of the Wuhan, Alpha, Delta and Kappa variants before (pre-Vacc) and after vaccination with the first dose (PD1) and the second dose (PD2) of BNT/Pfizer. The normalized S/EGFR ratio for reactivity with the Wuhan and Delta variants, and the S/GFP ratio for reactivity with the Alpha and Kappa variants was measured as a quantitative calculation of IgG1 anti-S antibody content. Sera were all used at a 1:50 dilution. Box and whiskers plots to represent minimum and maximum values as well as the median, and the mean (the mean with a cross) are shown. *p<0.05; **p<0.01; ****p<0.0001 (one-way ANOVA test). **(B)** Relative reactivity of the sera tested in **(A)** against the Alpha, Delta and Kappa variants versus the Wuhan strain was calculated according to the ratio of S/EGFR or S/GFP ratios and plotted individually for each serum donor. ****p<0.0001 (one-way ANOVA test). “NS” means "not significant" in the context of statistical analysis.

The effect of vaccination on the relative recognition of the S proteins of the Alpha, Delta and Kappa VOC versus that of the Wuhan strain was followed in sera from volunteers of nursing homes who had recovered after infections in 2020 and 2021 and were later vaccinated with BNT (**Figure S6**). Serum samples were taken just before vaccination (pre-Vacc, n=46, **Figure 4**), 15-21 days after the first dose (PD1, n=49) and 4 weeks after the second dose (PD2, n=70) of the BNT vaccine.

The first dose of BNT resulted in a significant increase in antibody reactivity against the Alpha and Kappa VOCs but not against the Wuhan and Delta ones (**Figure 4A**). Compared to the pre-vaccination samples, the second dose of BNT (PD2) resulted in significantly increased antibody titers against the four VOCs, although the differences between antibody titers induced after PD1 and PD2 were not obvious for the Delta and Kappa ones (**Figure 4A**). Indeed, the relative reactivity of IgG1 antibodies to the Alpha, Delta and Kappa VOCs compared to that of the Wuhan strain, was significantly reduced after the second dose of BNT compared to the first one (PD1 vs PD2, **Figure 4B**). These results support the data shown in **Figure 2C** suggesting that booster vaccination results in a relative loss of recognition of the Alpha, Delta, and Kappa VOC.

**Figure 5:**
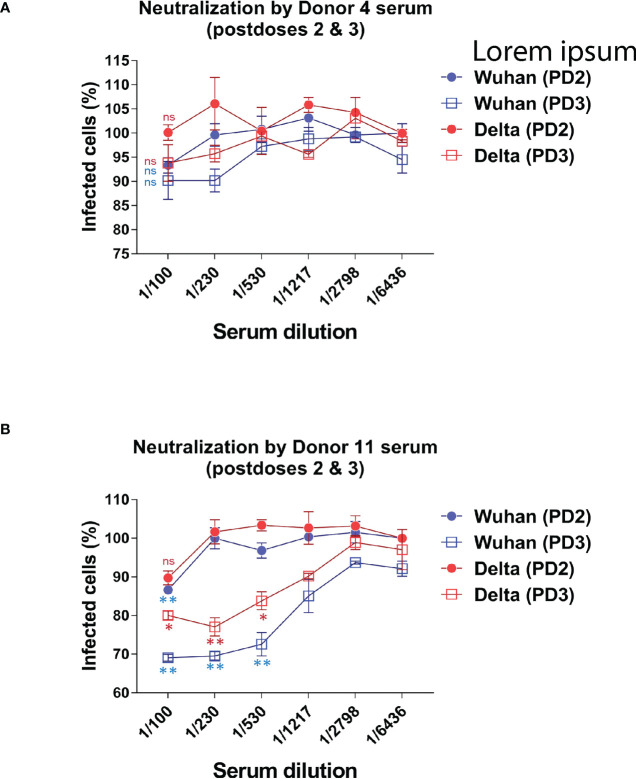
Evaluating the ability to neutralize the Wuhan and Delta strains after the second and third doses. The neutralizing capacity of sera from certain volunteers who received the second and third booster of the BNT-Pfizer vaccine was evaluated by comparing their ability to neutralize a GFP+ reporter lentivirus pseudotyped with the S protein of either the Wuhan or Delta strains. The results showed a decrease in the neutralizing capacity against the Delta variant after the third dose compared to the second dose. **(A)** The neutralizing capacity results against the Wuhan and Delta variants after the second and third dose of BNT are presented for patients #4. **(B)** The neutralizing capacity results against the Wuhan and Delta variants after the second and third dose of BNT are presented for patients #11.

In addition to comparing the relative titers of sera against the Wuhan and VOCs after the prime and boost vaccinations, we have carried out a small pilot assay to test the neutralization capacity of PD2 and PD3 (after a second booster dose) sera from two volunteers vaccinated with BNT. Using lentiviral particles expressing GFP and pseudotyped with the S protein of the Wuhan or the Delta strains, we titrated the neutralization capacity of sera from the two volunteers according to the conversion of infected ACE2+ HEK293 cells into GFP positive. Sera PD2 and PD3 from Donor#4 barely neutralized the pseudotyped lentiviruses at 1/100 dilution after PD2 and PD3, suggesting that vaccination had not resulted in a clear gain in neutralization capacity for either of the two strains (**Figure 5**). On the other hand, sera from Donor #11 showed increased neutralization activity for the Wuhan strain after the second booster (PD3) compared to sera from the first booster (PD2), reaching significant effect at a 1/500 dilution (**Figure 5**), An improvement of neutralization activity was seen for the Delta strain after the second booster (**Figure 5**). However, the comparison of the neutralization curves for Wuhan and Delta strains after the second booster showed that donor#11 had less capacity to neutralize Delta, indicating that the repeated vaccination with the same vaccine, based on the Wuhan sequence, boosts neutralization activity for the original isolate more strongly than for newer isolates.

## Discussion

In this study we have measured the production of antibodies of the IgG1 isotype to the spike protein of SARS-CoV-2 in response to the four different vaccines most frequently used in Spain. We have compared those data with antibodies present in sera from patients recovered from infection during the first wave of the COVID-19 pandemic in March-June 2020 and with sera from patients recovered from infection during the fourth wave between March-May 2021. Our results show that a priming first dose of the mRNA vaccines BNT/Pfizer and Moderna significantly induced a higher antibody response against the S protein of the Wuhan strain than the two adenovirus-based vaccines ChAd and Ad26, and superior to the titers found in the cohorts of 2020 and 2021 patients. The booster immunization with a second dose did not increase the average titer of antibodies except for the ChAd vaccine. The booster immunization with ChAd resulted in antibody titers that were similar to those generated in response to the booster immunization with Moderna or to the patient cohorts of 2020 and 2021, but still lower than the titers generated in response to the booster immunization with BNT. Therefore, in summary, our results indicate that two doses of the ChAd, BNT and Moderna vaccines are able to induce antibody titers against the original Wuhan strain equally or even higher to those found in post-convalescent patients. However, the message is different when we compared the relative reactivity of the antibodies against the Alpha, Delta and Kappa variants versus the Wuhan strain. Although antibodies made in response to vaccines based on the original Wuhan strain sequence do also bind the other three variants, (suggesting that most epitopes are conserved between the vaccine and the variant sequences), there is a relative loss of reactivity with the three VOCs compared to the Wuhan strain occurring upon administration of the booster dose of vaccine. This is somehow expected since repeated immunization with the same antigen sequence leads to the generation of higher affinity antibodies that fit better the epitopes of the immunogen. This increase in affinity has the negative side effect of reducing the “breadth” of the antibodies, that is, their capacity to bind to epitopes that differ slightly from those of the immunogen. Such effect of generating high-affinity antibodies that are however strain-specific has been widely documented in the search of broadly neutralizing antibodies for the human immunodeficiency virus (HIV) (28, 29). The long-term struggle, and still unsuccessful, to generate a vaccine that prevents infection by the tremendous diversity of clades and mutants of HIV, with the general conclusion that the tested vaccines are effective to prevent infection by the strain used for immunization but not by the myriad of variants found in the field (22). Another example is the result of vaccination with inactivated influenza virus isolated in immunization campaigns, that result in an efficient neutralization of that particular seasonal variant of influenza but results in reduced capacity to neutralize other variants (17, 30, 31). By contrast, antibody responses to natural infection are broad and exhibit different immunodominance patterns (32, 33). Another example of the different breadth in the antibody responses elicited by natural infection versus vaccination is in regard to COVID-19: a broad and sustained polyantigenic immunoreactivity against the S protein and other viral proteins has also been found in COVID-19 patients, in this case associated to the severity of symptoms (31). Our follow-up of the cohort of residents in nursing homes that were vaccinated with two doses of BNT shows that the two doses of vaccine increased the titers of anti-S antibodies against all the four variants. However, compared with the effect of the first dose, the second dose of vaccine did not result in further increases in the antibody titer against the Delta and Kappa variants and had the negative counterpart of reducing the relative reactivity of the antibodies with all three VOCs. This is relevant since the Alpha variant first and the Delta variant later, have been the most rapidly expanding VOCs in Europe and the USA in 2021 (https://www.cdc.gov/coronavirus/2019-ncov/science/science-briefs/fully-vaccinated-people.html) (31). Interestingly, a recent study using an assay similar to the Jurkat-S FC assay but based on the human B cell line Ramos, has also shown that booster vaccination with BNT increases the titer of anti-S antibodies against the Wuhan strain but not against the Alpha and Beta variants (25).

The aim of this research was to assess the prevalence of IgG1 against SARS-CoV-2’s S protein, given its high abundance in human blood and strong effector capabilities. The engagement and effector functions of IgG1 and IgG3 subclasses are crucial, with increased levels correlating to better prognosis and less severe infections caused by SAR-CoV2 (32, 33). Furthermore, IgG4 responses were predominantly found in individuals who received mRNA vaccines, whereas those vaccinated with adenovirus-based vaccines showed a lower IgG4 response (34, 35). Our results showing a loss of relative reactivity with the current VOCs suggest that third doses of the present vaccines, based on the Wuhan sequence, might not be the best approach to increase the immunity to the emerging VOCs. In our opinion, a third or even a fourth dose should be limited to the population that has been demonstrated to have been poorly responsive to the first two prime and boost doses and not to the general population. We believe a follow-up study with sera of people having received 3 or 4 doses of the vaccines would be highly interesting if our predictions of losing general reactivity against variant strains are correct.

## Data availability statement

The original contributions presented in the study are included in the article/**Supplementary Material**. Further inquiries can be directed to the corresponding author.

## Ethics statement

The studies involving human participants were reviewed and approved by Research Ethics Committee (no. #2352). The patients/participants provided their written informed consent to participate in this study.

## Author contributions

LH performed experiment, analyzed the data and designed research; PD performed research and analyzed the data; IB helped with experimentation; SR-P, MQ, RL-G, PA-V, ML, EP-A, SÁ, and AO provided clinical samples and data and revised the manuscript; MF and HS analyzed data and supervised research, BA supervised and designed research, analyzed data and wrote the manuscript. All authors contributed to the article and approved the submitted version.

## Supplementary material

Supplementary Figure 1 Conversion between normalized ratio and WHO standards 21/234 (Wuhan-1). The international WHO working standard 21/234 was used to obtain a calibration between the normalized S/EGFR MFI ratio on Jurkat-S(Wuhan) cells and Binding Antibody Units (BAU). The datapoints were adjusted to a sigmoidal 4PL fit, where X is in log10 (concentration) with a R^2 =^ 0.9980.

Supplementary Figure 2 Alignment of the amino acid sequences of the S protein of the Wuhan, Alpha, Delta and Kappa variants of SARS-CoV-2. Amino acid mutations found in the Alpha, Delta or Kappa variants are shown in red bold type. Alpha has 5 amino acid replacements and two amino acid deletions compared to the reference Wuhan strain; Delta has 3 amino acid replacements and Kappa has 4. The sequence corresponding to the Receptor Binding Domain (RBD) is boxed. Alpha has just one amino acid (N to Y) replacement in the RBD, Kappa two (L to R and E to Q) and Delta one (L to R).

Supplementary Figure 3 Titration of the different Jurkat-S variant cell lines. Serial dilutions of the positive control serum (C+) used in , was tested on the 4 Jurkat-S cell lines to determine their response to decreasing concentrations of antibody. A pre-COVID human serum (dilution 1:50) was used as negative control (grey squares).

Supplementary Figure 4 Spike protein expression at the plasma membrane. Mid-plane optical sections of Jurkat-S cells expressing the indicated variants and stained with a mouse monoclonal anti-S protein of SARS-CoV-2 (in green) and a CD45-PE antibody (in red) as a plasma membrane marker. The cell nuclei were stained with DAPI.

Supplementary Table 1 Experimental data of Wuhan and Alpha variants.

Supplementary Table 2 Experimental data combining Wuhan, Alpha, Delta and Kappa variants.
