# Supplementary figures and images for: Decreased breadth of the antibody response to the spike protein of SARS-CoV-2 after repeated vaccination
Source: Front Immunol. 2023 Apr 3;14:1157263. doi: 10.3389/fimmu.2023.1157263 (PMC10111966; doi:10.3389/fimmu.2023.1157263)

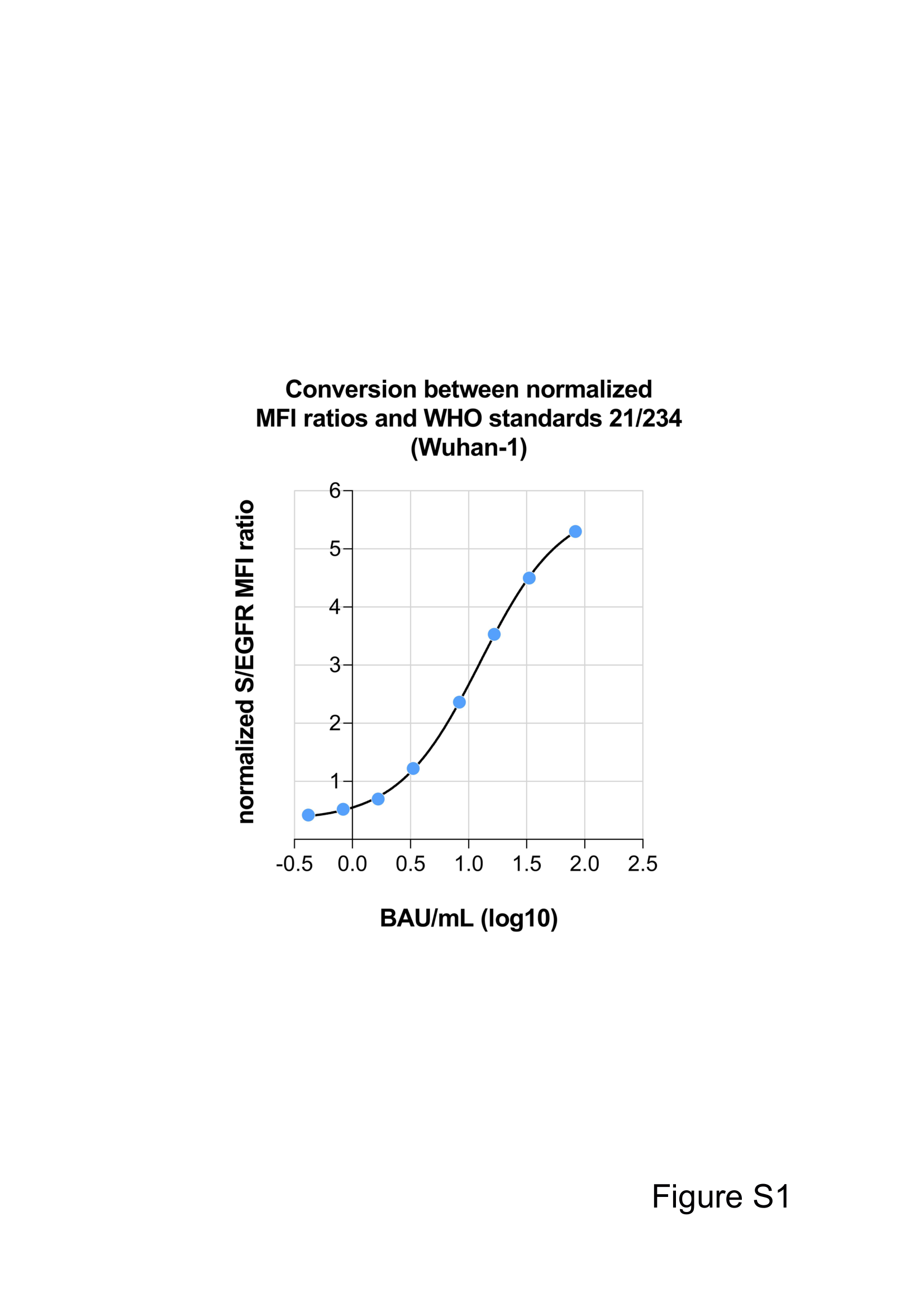

Supplement: Supplementary Figure 1 — Conversion between normalized ratio and WHO standards 21/234 (Wuhan-1). The international WHO working standard 21/234 was used to obtain a calibration between the normalized S/EGFR MFI ratio on Jurkat-S(Wuhan) cells and Binding Antibody Units (BAU). The datapoints were adjusted to a sigmoidal 4PL fit, where X is in log10 (concentration) with a R2 = 0.9980. [file Image_1.tiff]

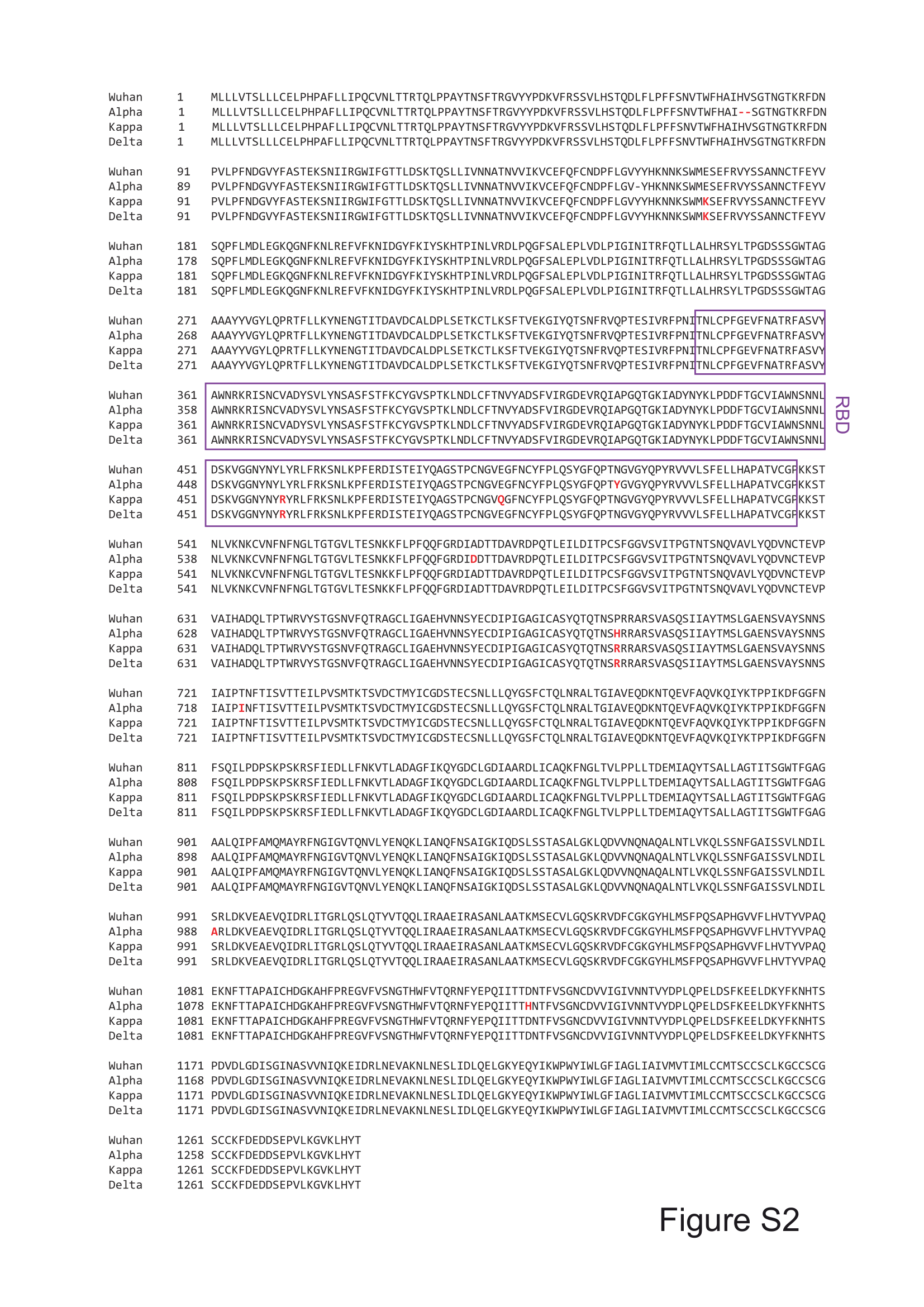

Supplement: Supplementary Figure 2 — Alignment of the amino acid sequences of the S protein of the Wuhan, Alpha, Delta and Kappa variants of SARS-CoV-2. Amino acid mutations found in the Alpha, Delta or Kappa variants are shown in red bold type. Alpha has 5 amino acid replacements and two amino acid deletions compared to the reference Wuhan strain; Delta has 3 amino acid replacements and Kappa has 4. The sequence corresponding to the Receptor Binding Domain (RBD) is boxed. Alpha has just one amino acid (N to Y) replacement in the RBD, Kappa two (L to R and E to Q) and Delta one (L to R). [file Image_2.tiff]

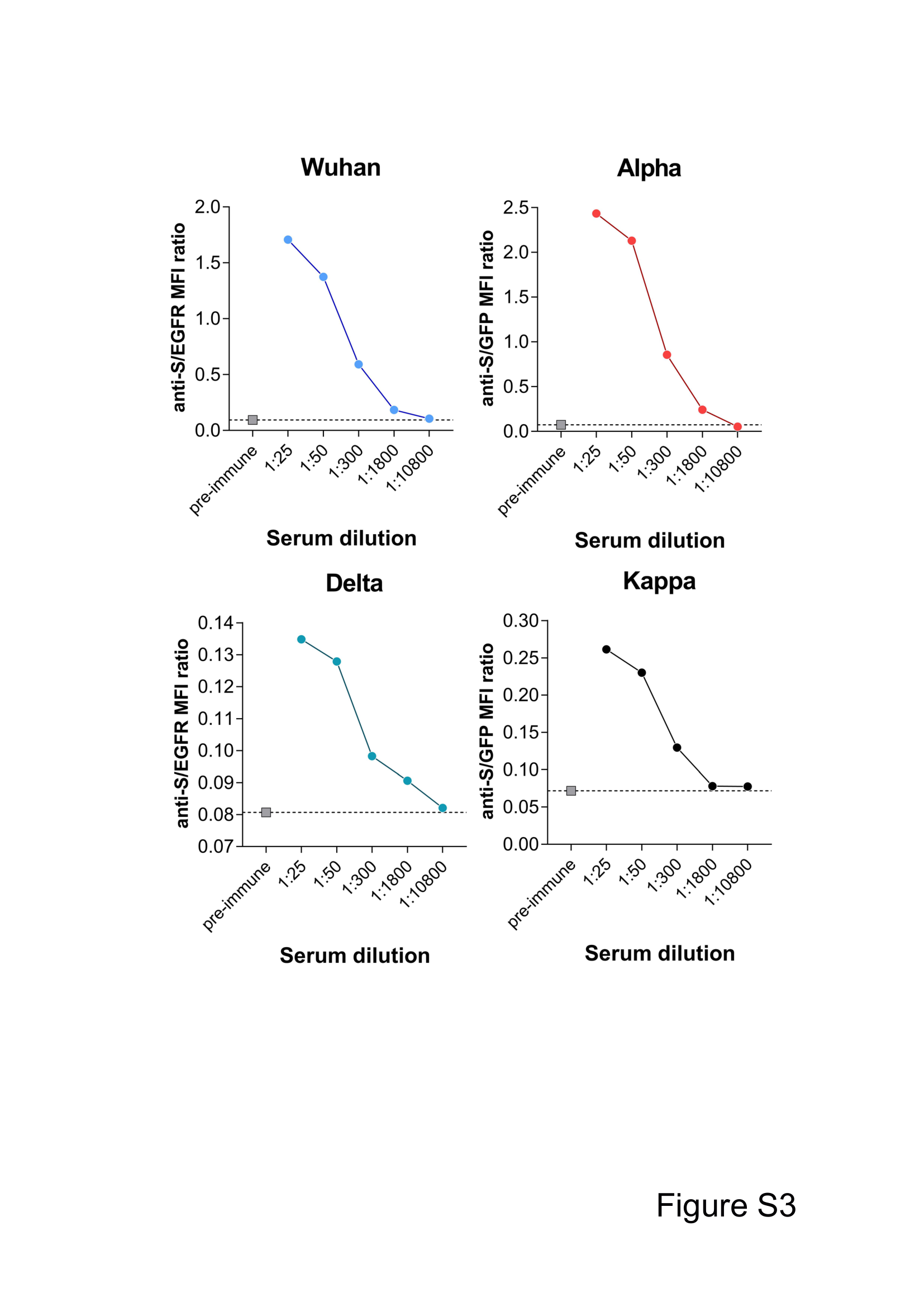

Supplement: Supplementary Figure 3 — Titration of the different Jurkat-S variant cell lines. Serial dilutions of the positive control serum (C+) used in , was tested on the 4 Jurkat-S cell lines to determine their response to decreasing concentrations of antibody. A pre-COVID human serum (dilution 1:50) was used as negative control (grey squares). [file Image_3.tiff]

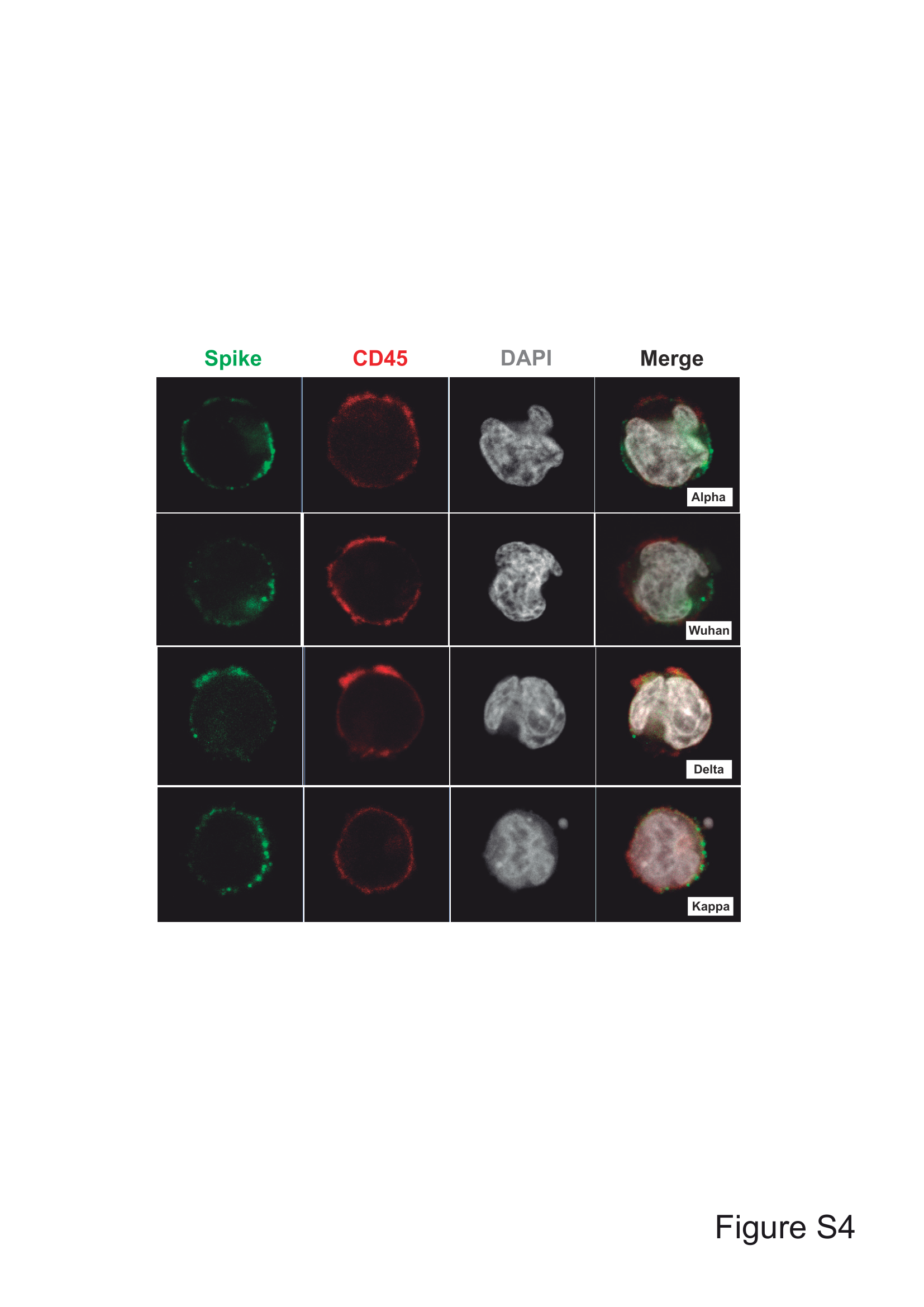

Supplement: Supplementary Figure 4 — Spike protein expression at the plasma membrane. Mid-plane optical sections of Jurkat-S cells expressing the indicated variants and stained with a mouse monoclonal anti-S protein of SARS-CoV-2 (in green) and a CD45-PE antibody (in red) as a plasma membrane marker. The cell nuclei were stained with DAPI. [file Image_4.tiff]
